# PHARE: a bioinformatics pipeline for compositional profiling of multiclonal *Plasmodium falciparum* infections from long-read Nanopore sequencing data

**DOI:** 10.1093/jac/dkae060

**Published:** 2024-03-19

**Authors:** Salome Hosch, Philipp Wagner, Johanna Nouria Giger, Nina Dubach, Elis Saavedra, Carlo Federico Perno, Jean-Chrysostome Gody, Marilou Sonia Pagonendji, Carine Ngoagouni, Christophe Ndoua, Christian Nsanzabana, Ulrich Vickos, Claudia Daubenberger, Tobias Schindler

**Affiliations:** University of Basel, Petersplatz 1, 4001 Basel, Switzerland; Department of Medical Parasitology and Infection Biology, Swiss Tropical and Public Health Institute, Kreuzstrasse 2, 4123 Allschwil, Switzerland; University of Basel, Petersplatz 1, 4001 Basel, Switzerland; Department of Medical Parasitology and Infection Biology, Swiss Tropical and Public Health Institute, Kreuzstrasse 2, 4123 Allschwil, Switzerland; University of Basel, Petersplatz 1, 4001 Basel, Switzerland; Department of Medical Parasitology and Infection Biology, Swiss Tropical and Public Health Institute, Kreuzstrasse 2, 4123 Allschwil, Switzerland; University of Basel, Petersplatz 1, 4001 Basel, Switzerland; Department of Medical Parasitology and Infection Biology, Swiss Tropical and Public Health Institute, Kreuzstrasse 2, 4123 Allschwil, Switzerland; University of Basel, Petersplatz 1, 4001 Basel, Switzerland; Department of Medical Parasitology and Infection Biology, Swiss Tropical and Public Health Institute, Kreuzstrasse 2, 4123 Allschwil, Switzerland; Department of Microbiology, Ospedale Pediatrico Bambino Gesù, Piazza di Sant’Onofrio, 4, 00165 Roma, Italy; Department of Intensive Care, Pediatric University Hospital Centre of Bangui, Bangui, Central African Republic; Laboratory of Parasitology, Institut Pasteur of Bangui, Bangui, Central African Republic; Medical Entomology Unit, Institut Pasteur of Bangui, Bangui, Central African Republic; National Malaria Control Program, Ministry of Health, Bangui, Central African Republic; University of Basel, Petersplatz 1, 4001 Basel, Switzerland; Department of Medical Parasitology and Infection Biology, Swiss Tropical and Public Health Institute, Kreuzstrasse 2, 4123 Allschwil, Switzerland; Department of Microbiology, Ospedale Pediatrico Bambino Gesù, Piazza di Sant’Onofrio, 4, 00165 Roma, Italy; University of Basel, Petersplatz 1, 4001 Basel, Switzerland; Department of Medical Parasitology and Infection Biology, Swiss Tropical and Public Health Institute, Kreuzstrasse 2, 4123 Allschwil, Switzerland; University of Basel, Petersplatz 1, 4001 Basel, Switzerland; Department of Medical Parasitology and Infection Biology, Swiss Tropical and Public Health Institute, Kreuzstrasse 2, 4123 Allschwil, Switzerland

## Abstract

**Background:**

The emergence of drug-resistant clones of *Plasmodium falciparum* is a major public health concern, and the ability to detect and track the spread of these clones is crucial for effective malaria control and treatment. However, in endemic settings, malaria infected people often carry multiple *P. falciparum* clones simultaneously making it likely to miss drug-resistant clones using traditional molecular typing methods.

**Objectives:**

Our goal was to develop a bioinformatics pipeline for compositional profiling in multiclonal *P. falciparum* samples, sequenced using the Oxford Nanopore Technologies MinION platform.

**Methods:**

We developed the ‘Finding *P. falciparum* haplotypes with resistance mutations in polyclonal infections’ (PHARE) pipeline using existing bioinformatics tools and custom scripts written in python. PHARE was validated on three control datasets containing *P. falciparum* DNA of four laboratory strains at varying mixing ratios. Additionally, the pipeline was tested on clinical samples from children admitted to a paediatric hospital in the Central African Republic.

**Results:**

The PHARE pipeline achieved high recall and accuracy rates in all control datasets. The pipeline can be used on any gene and was tested with amplicons of the *P. falciparum* drug resistance marker genes *pfdhps*, *pfdhfr* and *pfK13*.

**Conclusions:**

The PHARE pipeline helps to provide a more complete picture of drug resistance in the circulating *P. falciparum* population and can help to guide treatment recommendations. PHARE is freely available under the GNU Lesser General Public License v.3.0 on GitHub: https://github.com/Fippu/PHARE.

## Introduction

Malaria is one of the top three infectious diseases globally, with an estimated 247 million cases reported in 2021 and continuing efforts are needed for its control.^1^ Artemisinin combination therapy (ACT) is the primary treatment for *Plasmodium falciparum* (*Pf*), the deadliest malaria species. ACT consists of an artemisinin derivative combined with one of six partner drugs or drug combinations.^2,3^ Delivered together, the fast-acting artemisinin component rapidly kills most of the asexual blood stage parasites within a few days, and the longer-acting partner drug clears the residual parasite populations.^2,4^ Recent reports have confirmed the increased prevalence of *Pf* strains showing reduced clearance rate after ACT treatment indicative of partial resistance development in Africa.^5^ Modelling of widespread resistance to both artemisinin and a partner drug in Africa indicated a potential scenario of 16 million additional annual malaria cases^6^ resulting in nearly 80 000 additional malaria deaths each year, in addition to economic losses of 1 billion US dollars.^7^

Adaptations of guidelines for treatment with antimalarial drugs are developed based on parasitological data determined in therapeutic efficacy studies.^8,9^ Alternatively, sensitivity to antimalarial drugs can be assessed *in vitro* with IC_50_ studies^10^ and sensitivity to artemisinin by performing ring stage survival assays.^11^ However, since both *in vitro* and *in vivo* studies are expensive and labour-intensive, molecular markers, such as SNPs and copy number variations, can be used as indicators of resistance to a particular antimalarial drug.^12,13^ These markers can be identified through rapid and relatively inexpensive molecular biology techniques such as amplicon sequencing^14^ and quantitative PCR-based technologies.^15,16^ Incorporating blood sampling and storage on filter papers enables the analysis of samples from remote settings for the presence of drug resistance markers.^17,18^ Using molecular markers may be more appropriate for approaches where samples are collected regularly and analysed more frequently over a longer time period to monitor potential changes in drug resistance patterns over time.^19^ Longitudinal monitoring of antimalarial drug efficacy supported with molecular surveillance on resistance markers is essential for making data-driven decisions on combination therapy guidelines in a country or region.^20,21^

Malaria infections often consist of multiple parasite clones, known as multiclonal infections. Multiclonal malaria infections are common in areas with high malaria transmission rates^22^ and can complicate the identification of molecular markers, especially if some clones are present at lower densities within a sample. Compositional profiling aims to characterize the different clones present in the infection and their relative proportions.

Advances in single-molecule sequencing technologies and bioinformatics analyses have improved the ability to detect and analyse genetic variation within the parasite population, enabling the identification of molecular markers even in multiclonal infections.^23–25^ Additionally, using novel long-read sequencing methods such as Oxford Nanopore Technologies (ONT) MinION, full-length genes can be sequenced in one read. All SNPs present in this gene can therefore be analysed as one haplotype instead of SNP sites being analysed individually. However, most bioinformatics tools for haplotype calling have been developed for Illumina short read data and are difficult to adapt to single-molecule long-read sequencing data derived from ONT sequencing. Therefore, we developed the ‘Finding *P. falciparum* haplotypes with resistance mutations in polyclonal infections’ (PHARE) pipeline, which is designed to identify the molecular markers of drug resistance of all detectable clones in a sample, leveraging the technological advancements made available through long-read sequencing.

## Materials and methods

### Description of datasets

DNA extraction, PCR amplification, Sanger and ONT sequencing are described in the Supplementary Methods, primers are listed in Table S2 (available as Supplementary data at *JAC* Online).

To generate the first control dataset, the full-length dihydrofolate reductase (*pfdhfr*), dihydropteroate synthase (*pfdhps*) and kelch 13 (*pfK13*) genes of laboratory strains HB3, NF54 and K1 were amplified and ONT sequenced on a R10.4 flow cell. After basecalling, reads were length filtered and aligned to the respective reference sequence to filter out contaminants. Multiclonal samples were simulated by randomly selecting reads from two laboratory strains for each of the genes. The combinations NF54:HB3, NF54:Dd2 and HB3:Dd2 were analysed at 13 different mixing ratios, namely 1:99, 2.5:97.5, 5:95, 7.5:92.5, 10:90, 25:75, 50:50, 75:25, 90:10, 92.5:7.5, 95:5, 97.5:2.5 and 99:1.

For the second control dataset, Dd2 and NF54 culture media with highly similar parasitaemia (ΔCq 0.24 as determined by qPCR on the *P. falciparum* ribonucleotide reductase R2_e2 gene) were mixed to generate mixtures ranging from 100% NF54 to 0% NF54 (steps: 100%, 99%, 90%, 50%, 25%, 5%, 0.5%, 0%). The culture medium mixes were added to parasite-free human whole blood and DNA was extracted individually. The full-length *pfdhps* gene was amplified in three replicates for each mixture and sequenced on a R10.4 flow cell.

In a third control dataset to test the performance of detecting more than one clone in a single sample, DNA extracted from the *in vitro* control strains was mixed as follows: NF54-Dd2 (1:1), K1-HB3 (1:1) and a third sample with all four strains (1:1:1:1), followed by PCR amplification of the full-length *pfdhfr*, *pfdhps* and *pfK13* genes and sequencing on a R10.4 flow cell.

For the clinical dataset, dried blood spots obtained from febrile children at the Paediatric Hospital and University Complex of Bangui (CHUPB) in the Central African Republic were used. The full-length *pfdhfr*, *pfdhps* and *pfK13* genes of 12 *P. falciparum* positive samples were amplified and sequenced on a R9.4.1 flow cell. Amplification and Sanger sequencing of partial *pfdhfr*, *pfdhps* and *pfK13* genes was performed as a comparison.

### Data analysis and availability

Sanger sequencing data were analysed in SeqScape v.4.0 (Applied Biosystems). The sequences and basecalling were manually checked. Mutations were marked and a mutation report was generated. The mutation report was then saved as a .csv file and imported into R. ONT sequencing data was extracted as raw data in FAST5 format. Basecalling was done using the ‘super high accuracy’ basecalling in guppy (v.6.4.2). To test the impact of the guppy basecalling model, the ‘high accuracy’ and the ‘fast’ model were also tested with the second control dataset. FAST5 files were converted to POD5 files using the pod5 package (v.0.2.4) for basecalling in Dorado (v.0.4.2). All sequencing data was uploaded to the NCBI Sequence Read Archive under BioProject ID PRJNA974955.

### Programming of PHARE pipeline

The pipeline uses the tools Filtlong (v.0.2.1),^26^ minimap2 (v.2.24),^27^ samtools (v.1.16.1)^28^ and pysam (v.0.20.0).^29^ The code is written in bash, R (v.4.2.2) and python (v.3.7.11).

## Results

We developed PHARE, a novel multi-step bioinformatics pipeline to comprehensively profile the composition of genetic drug resistance markers in multiclonal samples. To fine tune parameters and validate the PHARE pipeline, we analysed a total of four datasets. The PHARE pipeline is documented and available for download and modification under the GNU Lesser General Public License v.3.0 on GitHub: https://github.com/Fippu/PHARE.

PHARE is a multi-step pipeline combining (i) quality control, (ii) SNP identification and (iii) graphical representation of haplotypes. Adjustable parameters and required reference files are listed in Table S1.

- The first step of the pipeline is to filter the input reads based on read quality and read length using Filtlong. Following this, an alignment to the provided reference sequence is done using minimap2.
- We developed a python-based algorithm that reduces the complexity of the data, by first defining SNPs, then discarding the remaining information of the sequenced read. This approach improves the analysis of error prone ONT reads. SNPs are found by iterating over the alignment and finding nucleotide sites, where at least two different bases are found with a frequency above a predefined threshold (SNP selection cut-off parameter, default 10%) or where most bases differ from the nucleotide provided in the reference.

ONT has higher error rates in low complexity regions since the translocation speed of DNA through the Nanopore is not constant and the basecaller cannot accurately determine the length of homopolymers where the electrical signal remains the same.^30^ During alignment, minimap2 introduces gaps at the ends of these homopolymers to correct for the erroneous reads.^31^ This leads to a lower coverage and a higher error rate at these sites. Therefore, SNPs at sites below a coverage minimum of 80% are filtered out.

In an optional step, silent mutations which do not affect the amino acid sequence, are removed. When investigating phenotypic variations such as drug resistance, silent SNPs are of limited interest and can reduce the yield of the pipeline, since every read must fulfil a minimal quality score (minqual parameter, default Phred score of 15) at each SNP site. If at any SNP site of a read a gap is present or the quality score at that position is below the threshold, the read is excluded from analysis. This leads to a linear decline in the number of reads passing quality control when adding SNPs at random sites (Figure S1).

The core step of the PHARE pipeline is the extraction of haplotypes, defined here as a set of genetic determinants located on a single gene, from each read. Like the detection of SNP sites, this is an iterative approach. The algorithm loops through all reads which did align to the reference and concatenates all the SNP sites to result in one haplotype per single read. In this step, reads with gaps at any of the SNP sites and reads with insufficient base quality are removed from further analysis. The output of the pipeline is a tab-separated file for each sample, which lists all present haplotypes and their respective read counts.

(iii) The tab-separated files with the results of the PHARE pipeline for each sample are analysed in an R script to generate a visual output of the data. In this step, the minimum haplotype frequency filter (default 5%) is applied, to filter out low abundance haplotypes that are considered noise.

### PHARE can identify SNPs with 100% accuracy and recall

**Figure 1.**
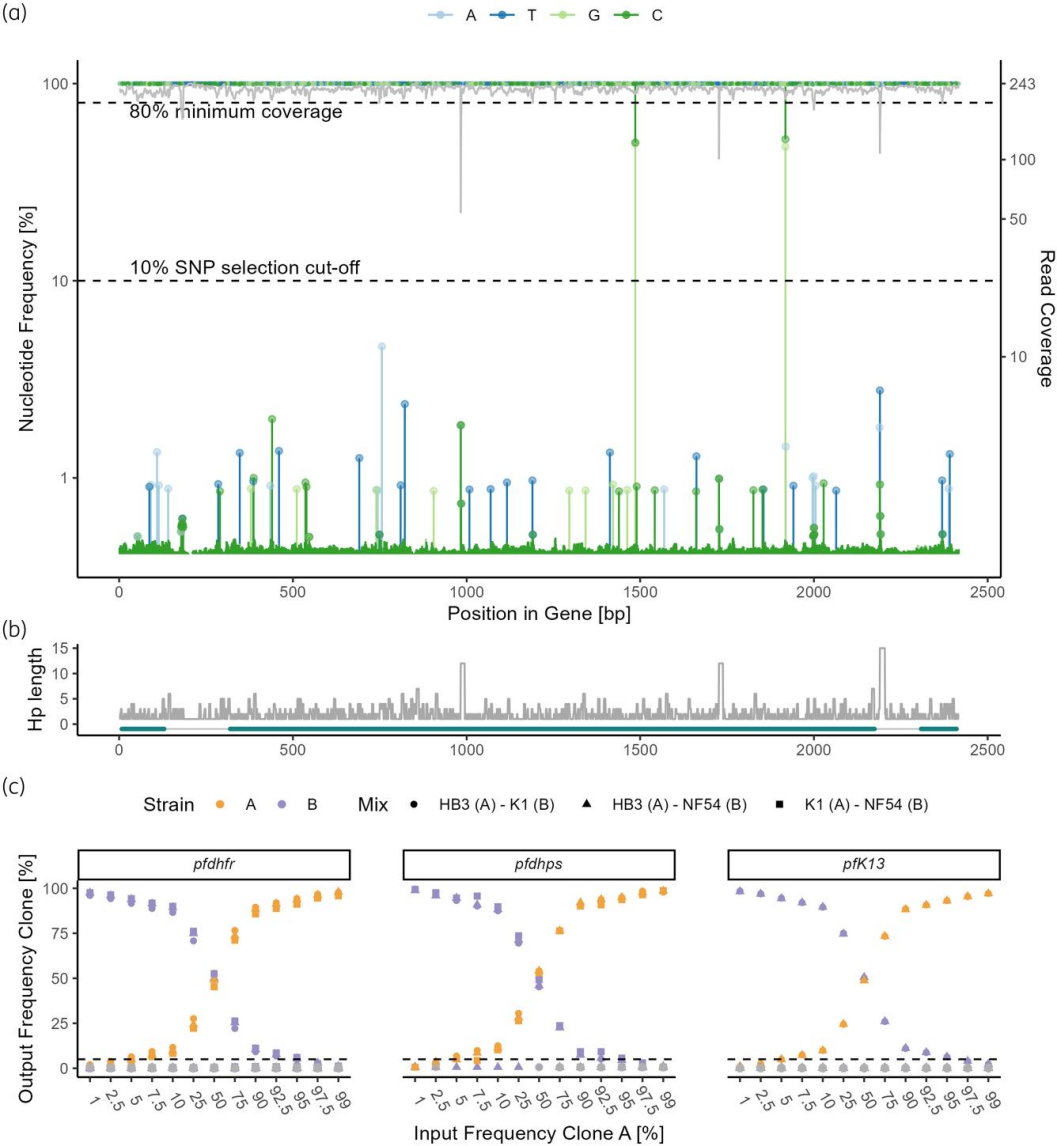
Simulated multiclonal infections were created by mixing reads from laboratory strains HB3, K1 and NF54 at different ratios ranging from 1% to 99%. (a) SNP calling in *pfdhps* with a mixture of 50% HB3 and 50% K1 reads. The nucleotide position is on the *x*-axis. The relative frequency of different bases is shown in the respective colour. The read coverage is shown as a grey line. The lower dotted line is the SNP selection cut-off, which was set at 10%. All positions where a nucleotide differs from the reference with a frequency above this threshold are considered SNP sites. The upper dotted line is the minimum coverage, which was set at 80%. Detected SNP sites where the coverage is below this threshold are excluded from further analysis. (b) The homopolymer length over the whole gene based on the reference sequence is shown in grey. Below are the exons (thick dark-green lines). (c) The input frequencies are compared against the output frequencies of the plot. A minimum haplotype frequency of 5% (dotted black line) was set to distinguish true haplotypes from noise (grey). This figure appears in colour in the online version of *JAC* and in black and white in the print version of *JAC*.

To tune the parameters and test the limits of detection of the PHARE pipeline we prepared simulated multiclonal samples from the first control experiment consisting of data from three *in vitro* culture strains with mixing ratios ranging from 1:99 to 99:1. In addition to the limits of detection, the SNP finding process was also validated on this simulated dataset with a total of 39 samples. A SNP selection cut-off of 10% was used. SNPs in the *pfdhfr*, *pfdhps* and *pfK13* genes were correctly identified in all samples, resulting in 100% accuracy and recall. Figure 1(a) is a graphical representation of the nucleotide distribution and SNP finding process in *pfdhps*, using the 50% HB3 and 50% K1 mixture sample: At two nucleotide positions, one of the minority nucleotides was more frequent than the SNP selection cut-off of 10% and these positions got correctly identified as SNPs. As explained in the pipeline description, the number of identical nucleotides cannot be determined accurately in homopolymers, leading to a dip in coverage below the read coverage minimum of 80% (Figure 1a). This can be observed in the three homopolymer regions of the *pfdhps* gene starting at nucleotide positions 983, 1727 and 2190, respectively (Figure 1b). By using a minimum haplotype frequency of 5%, the minor clone could be distinguished from noise, hence no false haplotypes were identified in all three genes (Figure 1c). Furthermore, we investigated the minimal coverage necessary to obtain accurate results (Figure S2). Below 224 reads, an increase in false haplotypes, which are still below the minimum haplotype frequency of 5%, can be observed.

### The PHARE pipeline performs better with more accurate basecalling models and can distinguish true haplotypes from sequencing errors

**Figure 2.**
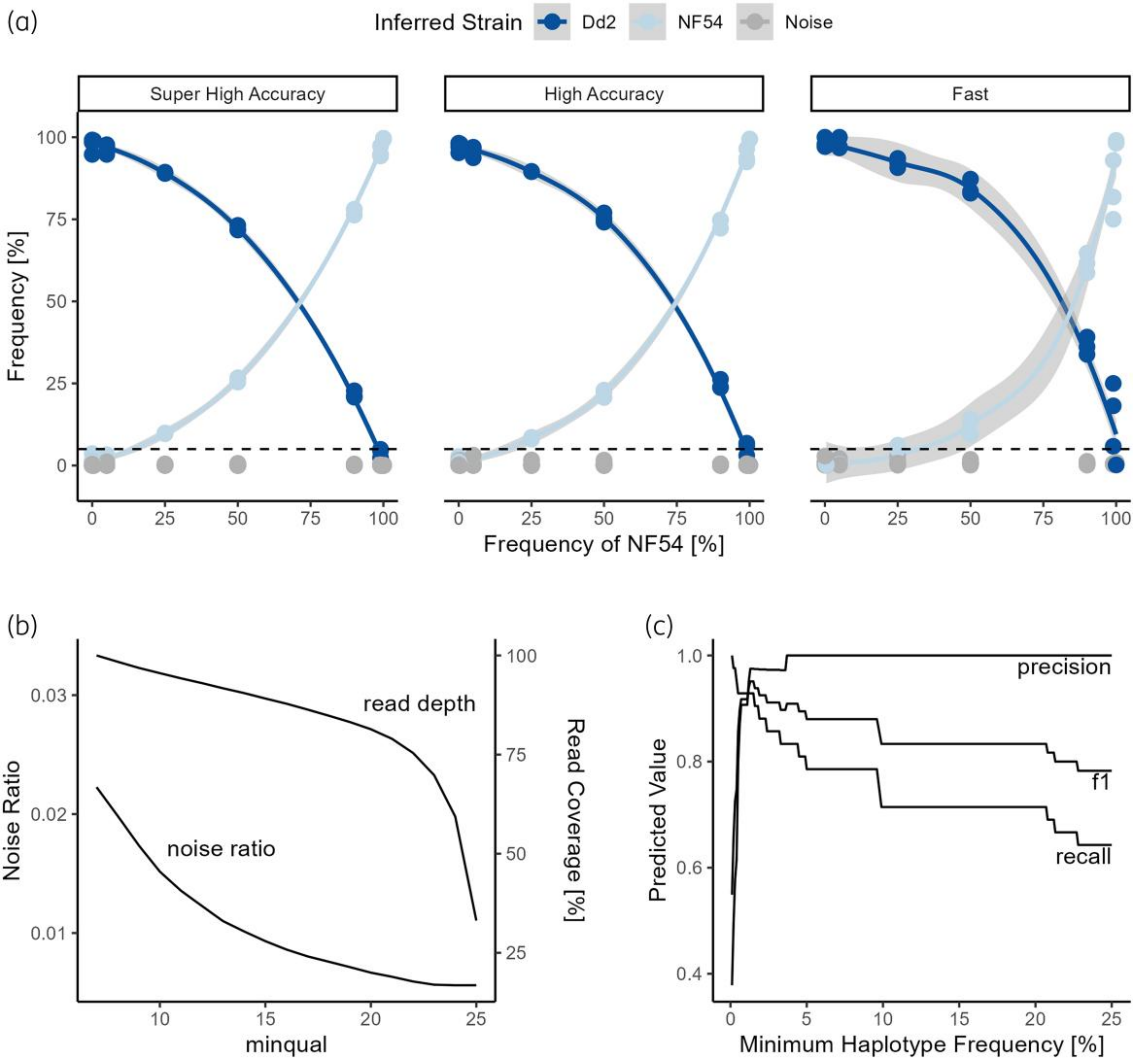
Data from *pfdhps* experiment with NF54 and Dd2 *in vitro* culture strains at different mixing ratios. (a) Using a minimal quality score of 15 and three different basecalling models. The colours indicate the strain that was inferred based on the haplotypes found by the PHARE pipeline [436f/613s (fs) in Dd2 and S436/A613 (SA) in NF54]. Grey dots represent additional haplotypes that are considered sequencing artefacts. A minimum haplotype frequency of 5% (dotted black line) was set to distinguish true haplotypes from noise. (b) Read coverage and noise ratio (the fraction of noise reads as part of the total amount of reads) at different minimum read qualities (Phred scores). (c) Precision, recall and f1 score with the minqual parameter set to 15. Both (b) and (c) use data generated by ‘super high accuracy’ basecalling. This figure appears in colour in the online version of *JAC* and in black and white in the print version of *JAC*.

In the second control experiment, culture media of two *in vitro* culture strains (NF54 and Dd2) with highly similar parasitaemia were mixed at different concentrations. *Pfdhps* was amplified in three technical replicates and ONT sequenced to test the impact of the guppy basecalling model on the pipelines output and to test the quantitative performance and limit of detection of the PHARE pipeline. The performance of the PHARE pipeline improves with more accurate basecalling (Figure 2a). Haplotypes could be recalled with a high accuracy and low variability when using the ‘super high accuracy’ basecalling. The three replicates had very little variation (IQR 0.95%) and haplotypes of the laboratory strains 436f/613s (Dd2) and S436/A613 (NF54) were correctly identified. No false haplotypes were observed using the ‘super high accuracy’ basecalling. Variation between replicates increased when changing the basecalling model to ‘high accuracy’ and even more when using the ‘fast’ basecalling model. Furthermore, SNP calling could not be reliably done using the ‘fast’ basecalling dataset, since too much noise was masking the true SNPs and the coverage at many nucleotides was below 80% (Figure S3). Therefore, the same SNPs that were found in the ‘super high accuracy’ data were manually set for the ‘fast’ basecalling in this experiment. All subsequent analyses were performed using ‘super high accuracy’ basecalling. The PHARE pipeline was also tested with data where the basecalling had been performed with Dorado. Results obtained with Dorado basecalled data (Figure S4) were highly similar to the results obtained with guppy basecalled data (Figure 2a), with ‘fast’ basecalling insufficient for SNP calling.

Over all concentrations, the percentage of reads mapping to the NF54 haplotype was lower than the frequency of NF54 in the original sample. To determine whether this was caused by bias in the PHARE pipeline or skewed sequencing data, we compared the results generated with PHARE to mapping the full-length reads directly to the *pfdhps* reference sequences of the two laboratory strains NF54 and Dd2 with minimap2 and calculating the relative frequencies of reads mapping to each reference sequence. The comparison between the relative frequencies of haplotypes identified by PHARE and those of reads mapped to a predefined reference sequence by minimap2 reveals a strong correlation between both methods, with a *R*^2^ > 0.99 (Figure S5). This implies that the observed discrepancies in haplotype frequencies as identified by PHARE and the frequency of NF54 in the original sample may not be attributed to the PHARE pipeline. Instead, it suggests the influence of external factors, such as sequencing biases or random variations during sample preparation, which could affect the distribution of reads observed in the analysis.

We determined the impact of the minqual parameter of the pipeline that sets both the minimal quality that must be reached at the SNP sites as well as the minimal quality of the whole read that was assigned during basecalling. The minimal quality is estimated by the guppy basecaller and corresponds to a Phred score *Q*, which is defined as *Q* = −10 × log_10_(*P*) where *P* is the sequencing error probability. As an example, a Phred score of 10 corresponds to 10% error probability. The effect of the minqual parameter on the ratio of noise haplotypes compared to true haplotypes and its influence on the read coverage is shown in Figure 2(b). Increasing minqual led to a reduction in noise, although the benefits were decreasing. Meanwhile the read coverage declined linearly with increasing minqual, with a rapid decline after minqual 20. We settled on a minqual of 15 for further analysis. We then assessed the effect of the minimum haplotype frequency that differentiates true haplotypes from false haplotypes on precision, recall and f1 score (Figure 2c). Precision reached its maximum at a minimum haplotype frequency of more than 3.6%, while the recall reached a plateau between 5.0% and 9.8%. We therefore decided on a minimum haplotype frequency of 5% for subsequent experiments.

### Compositional profiling of four haplotypes is possible with the PHARE pipeline

**Figure 3.**
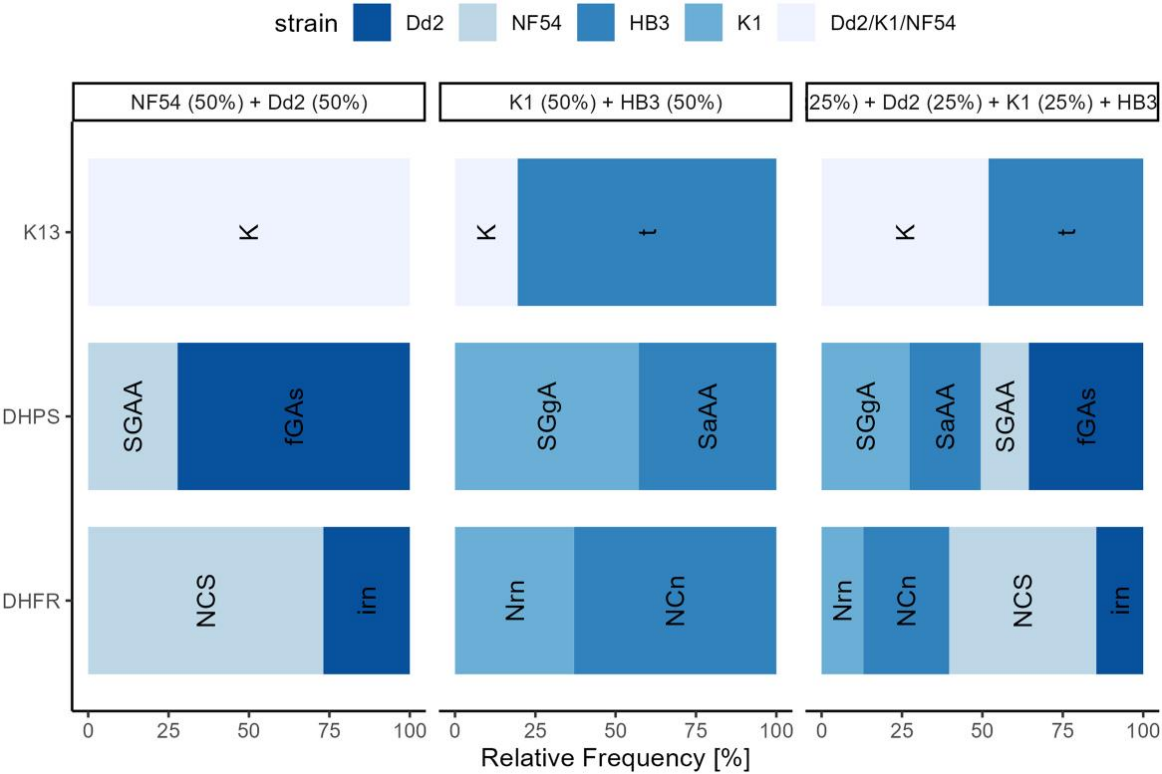
PHARE pipeline output for *pfdhfr*, *pfdhps* and *pfK13*. The widths of the bars indicate the relative frequency of the haplotype written on the bar as determined by the PHARE pipeline. The colours indicate the laboratory strain matching the haplotype found by the pipeline. SNP sites are N51i, C59r and S108n for *pfdhfr*; S436f, G437a, A581g and A613s for *pfdhps* and K189t for *pfK13*. This figure appears in colour in the online version of *JAC* and in black and white in the print version of *JAC*.

We tested the ability of the PHARE pipeline to detect more than two haplotypes in a single sample, using the three drug resistance marker genes *pfdhfr*, *pfdhps* and *pfK13*. DNA of the four *in vitro* culture strains NF54, Dd2, HB3 and K1 was mixed in equimolar ratios for two or four strains and amplified by PCR. Figure 3 shows the PHARE pipeline outputs for mixes of NF54 + Dd2, HB3 + K1 and all four culture strains combined. All expected haplotypes and no false haplotypes were detected. The estimated haplotype frequency did not always correspond to the input amount of DNA before PCR. For example, in the equimolar mixture of NF54 and Dd2, the haplotype corresponding to NF54 was found with a relative frequency of 67.3% in *pfdhfr* but only 27.6% in *pfdhps*. In *pfdhps* and *pfdhfr* the four haplotypes differ by one to three SNPs leading to an amino acid exchange, while in *pfK13* only HB3 differs from the other three strains by one single SNP making a distinction not always possible.

### The SNPs detected by the PHARE pipeline share a high agreement with conventional Sanger sequencing, while enabling the detection of complex haplotypes

Next, we used a sample set of 12 dried blood spots from the Central African Republic to understand the performance on clinical samples sequenced on ONT flow cell R9.4.1. Since older chemistry was used for sequencing, adapted parameters were used for SNP calling, namely the coverage minimum was set to 50%.

PHARE and Sanger sequencing detected the same three and five SNP sites in *pfdhfr* and *pfdhps*, respectively (Table 1). In *pfdhfr*, the same variants were detected for all 12 samples. At the G437a SNP site in *pfdhps*, PHARE and Sanger sequencing disagreed in three samples: the first sample was G437/437a with PHARE and 437a with Sanger, the second sample was G437/437a with PHARE and G437 with Sanger and the third sample was G437 with PHARE and G437/437a with Sanger. In *pfK13* both PHARE and Sanger found no SNPs in the 849 bp region analysed by Sanger sequencing. However, outside this region the K189n and K189t SNPs were found by PHARE.

**Table 1.** Comparison of drug resistance SNPs detected with Sanger and ONT sequencing for 12 clinical isolates from the Central African Republic

Marker	SNP	Agreement over 12 samples (%)		WT	Mutated	Mixed
*pfdhfr*	N51i	100		1	11	0
	C59r	100		0	12	0
	S108n	100		0	12	0
*pfdhps*	I431v	100		10	1	1
	S436a	100		2	6	4
	G437a	75	PHARE	5	2	5
			Sanger	5	3	4
	K540e	100		1	1	2
	A581g	100		11	0	1
*pfK13*	WT^a^	100		12	0	0

^a^Outside the 849 bp region analysed by Sanger, the K189n and K189t SNPs were found.

**Figure 4.**
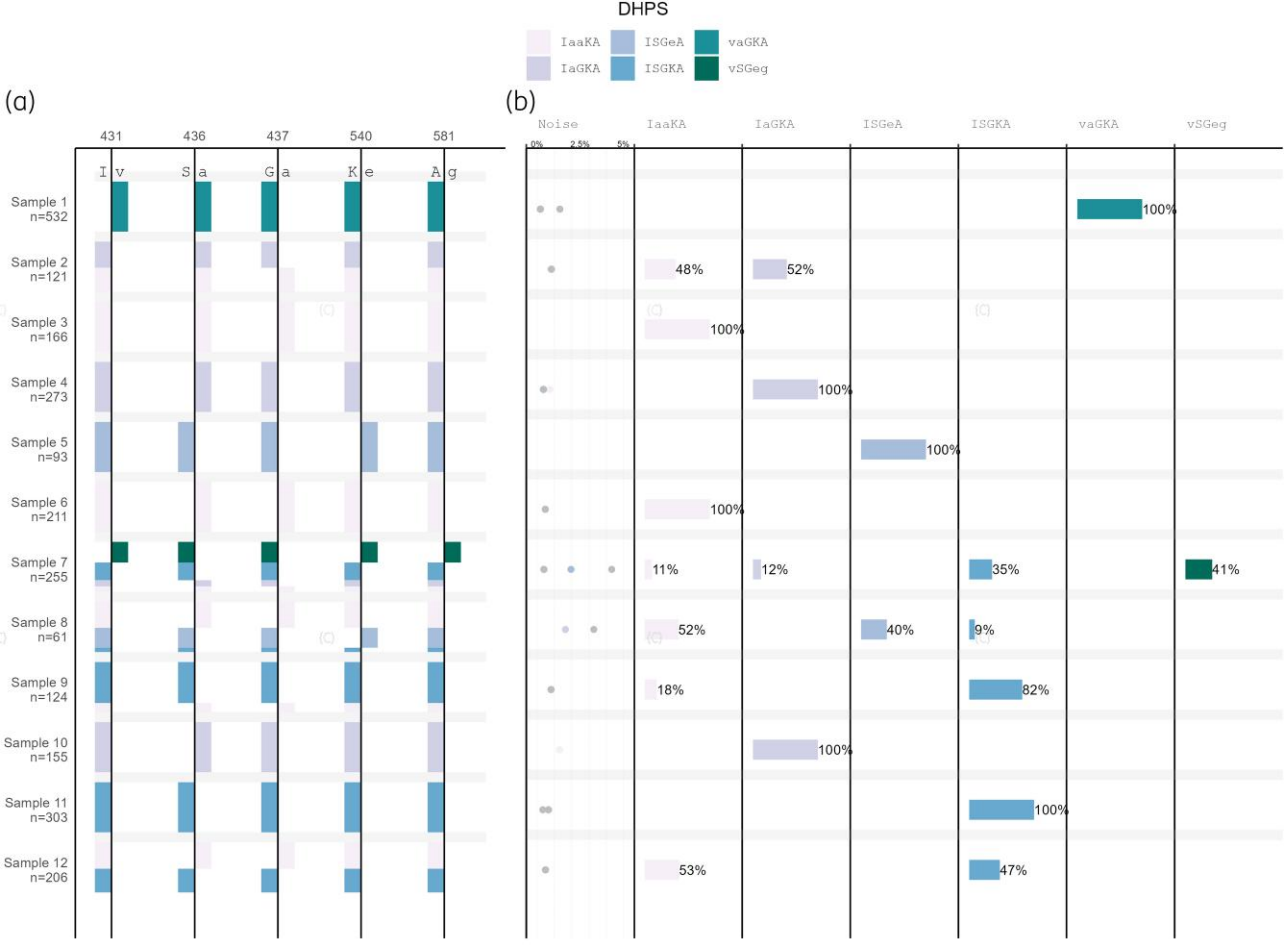
PHARE pipeline output for 12 clinical samples from the Central African Republic. (a) SNP sites and the relative frequencies of mutated and WT SNPs for each sample. (b) Relative frequencies of each haplotype per sample, as well as the noise level. Every point in the noise graph represents a hypothetical haplotype that was filtered out because it is below the minimum haplotype frequency. This figure appears in colour in the online version of *JAC* and in black and white in the print version of *JAC*.

To demonstrate the default output of the pipeline, the results for *pfdhps* are shown in Figure 4. Using the PHARE pipeline we were able to find six different *pfdhps* haplotypes based on five SNP sites. We found up to four haplotypes in a single sample. In *pfdhfr*, we found two different haplotypes based on N51i. Eleven of the 12 samples had the 51i mutated haplotype and one the N51 wild-type haplotype. The SNPs C59r and S108n SNPs were mutated in all samples. Furthermore, all SNP sites which were found by the PHARE pipeline in *pfdhps* and *pfdhfr* are known sites of drug resistance.

In *pfK13*, three different haplotypes were found, with one to three haplotypes per sample based on K189, which was mutated to either threonine or asparagine. As opposed to *pfdhps* and *pfdhfr*, the SNP found by the PHARE pipeline in *pfK13* in this clinical sample set has not been reported to be associated with drug resistance.

### PHARE provides additional information compared to other available tools for ONT reads

During the preparation of this paper, a study using ONT sequencing of malaria drug resistance markers in Ghana was published.^32^ The study used nano-rave (https://github.com/sanger-pathogens/nano-rave) for SNP calling and haplotype determination without analysing mixed infections. We applied our PHARE pipeline to their publicly available data, adapting it to their five gene targets. Our analysis of 196 samples was completed within 15 minutes. A comparison with nano-rave showed similar SNP calling results, but PHARE also identified mixed haplotypes and additional drug resistance-associated SNPs in the *pfdhps* gene, not covered by Girgis *et al.* (Figure S6), demonstrating PHARE’s enhanced analytical capabilities.

## Discussion

Here we present the development of the PHARE pipeline, a novel method for compositional profiling of haplotypes in multiclonal *Pf* positive samples. Our pipeline has shown excellent performance in both control and clinical datasets, detecting up to four clones in a sample and detecting minor clones with an occurrence of as low as 5%. With upcoming technical improvements of ONT, the sensitivity of the pipeline is expected to increase further. Against popular misconception, it is possible to analyse SNPs in ONT data. Numerous studies have shown the feasibility of using Nanopore data for SNP analysis^33,34^ and multiple tools have been developed for this purpose, such as Longshot,^35^ NanoSNP^36^ and Clair.^37^

While qualitative performance of the PHARE pipeline was excellent, the estimated haplotype frequency did not always correspond to the input amount of DNA before PCR. Bias during PCR is a possible cause and could be reduced with a lower number of PCR cycles as well as sufficient template DNA.^38^ Furthermore, while several publications state that ONT sequencing does not suffer from a GC content bias in terms of reads sequenced,^30,38–40^ others did find biases resulting from GC content and read length.^41^ Further investigation into the quantitative performance of the PHARE pipeline is needed.

Differentiating real, well-described haplotypes from false haplotypes that occur due to sequencing errors is a trade-off between avoiding false positives and being sufficiently sensitive. To avoid false positives and reach a specificity of 100%, we used a minimum haplotype frequency of 5%, discarding haplotypes with a prevalence below. This limits the sensitivity and prohibits detecting minority clones with frequencies below 5%. Depending on the research question, a lower value could be used, increasing the sensitivity. Read coverage did not have a strong influence on the relative frequency of haplotypes reported by the pipeline when comparing replicates. However, the required sequencing coverage depends on the number of variable sites of the genetic marker. In our experience, around 10% of the raw reads pass all filtering steps and since the frequency of false haplotypes can increase below 224 reads post filtering, we suggest having at least 2240 raw reads. The beginning and end of ONT reads tend to be of lower quality than the middle section.^30,40^ Therefore, it is important to design amplicons long enough to accommodate for this, preferably with primer binding sites outside the gene of interest.

It is crucial to differentiate between samples containing one clone with a combination of drug resistance SNPs and samples with the same SNPs spread over multiple clones, because for some antimalarial resistance genes, drug resistance increases exponentially with the addition of further SNPs to the same gene.^42^ If the SNP sites responsible for generating malaria drug resistance are far apart on a single gene, it is necessary to sequence and analyse the full-length gene using long-read sequencing. This is where ONT sequencing combined with the PHARE pipeline offers significant benefits over other methods generating shorter reads such as Illumina < 600 bp^43^ and Sanger < 900 bp.^44^

A study using ONT data of *Pf* partial drug resistance markers using majority consensus calls was published during revision of the paper, and their sequencing data were made publicly available.^32^ The PHARE pipeline detected all SNP sites that were described in the publication with highly comparable results. In addition, mixed haplotypes could be detected. Analysing more samples and conducting further studies will provide a deeper and more refined understanding of its ability in compositional profiling of multiclonal *Pf* infections. We believe that the PHARE pipeline has important implications for tracking the spread of resistance against malaria drugs, especially since drug resistance markers are monitored in minority clones and across the whole gene without previous knowledge of SNP sites.

## Supplementary Material

dkae060_Supplementary_Data

## Supplementary data

Figures S1 to S6 and Tables S1 and S2 are available as Supplementary data at *JAC* Online.
