# Supplementary material for: PHARE: a bioinformatics pipeline for compositional profiling of multiclonal Plasmodium falciparum infections from long-read Nanopore sequencing data
Source: J Antimicrob Chemother. 2024 Mar 19;79(5):987–96. doi: 10.1093/jac/dkae060 (PMC11062946; doi:10.1093/jac/dkae060)
Supplement: dkae060_Supplementary_Data [file dkae060_supplementary_data.docx]

**Supplementary methods**

Control experiments using laboratory *P. falciparum* clones to validate the PHARE pipeline

*P. falciparum* *in vitro* culture strains Dd2, HB3, K1 and NF54 were used to develop and test our bioinformatics analysis pipeline. For each strain, the cultured parasites were mixed with *P. falciparum* negative human blood and extracted with the QIAamp DNA Blood Mini Kit (Qiagen) according to the manufacturer’s instructions. To generate the first dataset, the dihydrofolate reductase (*pfdhfr*), dihydropteroate synthase (*pfdhps*) and kelch 13 (*pfK13*) genes of strains HB3, NF54 and K1 were amplified and ONT sequenced on a R10.4 flow cell.

For the second control dataset, Dd2 and NF54 culture media with highly similar parasitaemia (ΔCq 0.24 as determined by qPCR on the *P. falciparum* ribonucleotide reductase R2_e2 gene ^1^) were mixed to generate mixtures ranging from 100% NF54 to 0% NF54 (steps: 100%, 99%, 90%, 50%, 25%, 5%, 0.5%, 0%). The culture medium mixes were added to parasite-free human whole blood and DNA was extracted individually. The *pfdhps* gene was amplified in three replicates for each mixture and sequenced on a R10.4 flow cell.

In a third control dataset to test the performance of detecting more than one clone in a single sample, DNA extracted from the *in vitro* control strains was mixed as follows: NF54-Dd2 (1:1), K1-HB3 (1:1) and a third sample with all four strains (1:1:1:1), followed by PCR amplification of the *pfdhfr*, *pfdhps* and *pfK13* genes and sequencing on a R10.4 flow cell.

SNP detection rate and performance of the PHARE pipeline compared to conventional methods using clinical samples

Blood samples were collected at the Paediatric Hospital and University Complex of Bangui (CHUPB) in the Central African Republic from children with fever between March 8^th^ and 13^th^, 2021. Routinely collected data included malaria rapid diagnostic test (RDT) results, thick blood smear microscopy and full blood count. Dried blood spots were collected and then sent to the Swiss Tropical and Public Health Institute (Swiss TPH) for analysis of antimalarial resistance markers. DNA extraction was performed using a previously established extraction protocol ^2,3^. To generate the clinical dataset, the *pfdhfr*, *pfdhps* and *pfK13* genes of twelve *P. falciparum* positive samples were amplified and sequenced on a R9.4.1 flow cell.

The study was conducted in accordance with the Declaration of Helsinki and was approved by the Ethics and Scientific Committee from the University of Bangui (approval n°3/UB/FACSS/CSCVPER/PER) and by the Ministry of Health of the Central African Republic (approval n°0277/MSPP/CAB/DGSPP/DMPM/ SMEE from 5 august 2002) as part of the communicable and endemic diseases surveillance diagnostic program. The patients were informed about the objectives of the study and nature of their participation. Then, written and signed informed consent was obtained from the parents on behalf of their children.

PCR amplification for Sanger sequencing

For the detection of antimalarial drug resistance markers *pfdhps,* *pfdhfr* and *pfK13*, previously established protocols were used ^4,5^. Primer sequences and PCR product lengths are listed in supplementary table 2. In brief, a primary PCR was performed with 5 µl DNA in a reaction volume of 25 µl, using 1 U HOT FIREPol DNA Polymerase (Solis Biodyne) and 0.25 µM primers. For *pfdhfr* and *pfdhps* primary PCRs, the following thermal cycling conditions were used: initial denaturation at 95 °C for 12 minutes, 30 cycles of 95 °C for 30 seconds, 52 °C for 90 seconds, 72 °C for 90 seconds, followed by final extension at 72 °C for 5 minutes. For *pfK13* primary PCR, the following cycling conditions were used: initial denaturation at 95 °C for 15 minutes, 30 cycles of 95 °C for 30 seconds, 58 °C for 2 minutes, 72 °C for 2 minutes, followed by final extension at 72 °C for 5 minutes. Nested PCRs were performed for each gene individually, using 1 µl DNA in a reaction volume of 50 µl, using 2 U HOT FIREPol DNA Polymerase (Solis Biodyne) and 0.25 µM primers. For *pfdhfr* nested PCR, the following thermal cycling conditions were used: initial denaturation at 95 °C for 12 minutes, 30 cycles of 95 °C for 30 seconds, 52 °C for 90 seconds, 72 °C for 90 seconds, followed by final extension at 72 °C for 5 minutes. For *pfdhps* nested PCR, the following thermal cycling conditions were used: initial denaturation at 95 °C for 12 minutes, 30 cycles of 95 °C for 30 seconds, 58 °C for 90 seconds, 72 °C for 90 seconds, followed by final extension at 72 °C for 5 minutes For pfK13 nested PCR, the following cycling conditions were used: initial denaturation at 95 °C for 15 minutes, 40 cycles of 95 °C for 30 seconds, 60 °C for 1 minute, 72 °C for 1 minute, followed by final extension at 72 °C for 5 minutes. The final PCR product was checked for correct amplification on the QIAxcel Advanced Instrument and analysed with the corresponding QIAxcel ScreenGel 1.6.0 software (Qiagen). Samples with no band in the expected size were repeated. The successfully amplified samples were then sent to Microsynth AG (Balgach, Switzerland) for Sanger sequencing using the same primers as the nested PCR.

PCR amplification, library preparation and ONT sequencing

The full-length drug resistance genes *pfdhfr*, *pfdhps* and *pfK13* were amplified separately using published primers ^6^ with a reaction concentration of 0.5 µM. PCR amplification was performed with 3 µl of template DNA in a reaction volume of 25 µl, using 1x KAPA HiFi HotStart ReadyMix (Kapa Biosystems) according to the manufacturer’s protocol. The conditions of the PCR were: 95 °C for 3 minutes, 35 cycles of 95 °C for 15 seconds, 50 °C for 30 seconds and 62 °C for 3 minutes, followed by final extension at 62 °C for 5 minutes. All PCR products were evaluated by agarose gel electrophoresis.

Amplified DNA was purified using 0.8x volume AMPure® XP beads (Beckman Coulter) and quantified with the Qubit dsDNA HS Assay Kit (Invitrogen). For the control datasets using laboratory strains, the Native Barcoding Kit 96 (ONT SQK-NBD112.96) and R10.4 flow cell (ONT FLO-MIN112) were used. For the clinical samples, Ligation Sequencing Kit (ONT SQK-LSK109), Native Barcoding Expansion 13-24 (EXP-NBD114) and R9.4.1 flow cell (ONT FLO-MIN106D) were used. Sequencing libraries were prepared according to manufacturer’s instruction. Briefly, 200 fmol amplicon DNA were end-prepped, unique barcodes were ligated, and sequencing adapters were ligated. The libraries were loaded onto the flow cell and sequenced on the MinION Mk1C.

**Supplementary Tables**

| Parameter | Description | Default value |
| --- | --- | --- |
| sample_dir | Directory where basecalled sequencing reads are stored in a single file per sample | data/samples |
| SNP_selection_cut-off | Minimum % of sequencing reads with SNP at this site | 10% |
| coverage_minimum | Minimum % of reads covering a nucleotide position considered a SNP | 80% for ONT sequencing chemistry R10.4 or 50% for ONT sequencing chemistry R9.4.1 |
| minimum_haplotype_frequency | Minimum % of reads with certain haplotype | 5% |
| minqual | Minimum Phred score of reads at SNP sites | 15 |
| minimal_number_of_reads_per_sample | Minimum number of reads required for haplotype analysis | 50 |
| Gene | Gene name |  |
| generef | Gene reference in fasta format |  |
| genbank_ref | genbank report corresponding to the gene reference file in .jsonl format |  |
| Contig | Name of the contig in the fasta file |  |
| Targetlen | Length of the PCR amplicon |  |
| Amplicon_start | In case the amplicon is shorter than the provided gene reference, the start and end positions of the amplicon relative to the generef .fastq file have to be provided.  If they are left empty, the pipeline assumes that the amplicon is longer than the target gene. | NA |
| Amplicon_end |  | NA |
| Minlen | Minimum length of reads | Targetlen - 50 |
| Maxlen | Maximum length of reads | Targetlen + 350 |

Table S1. Adjustable parameters and input files of the PHARE pipeline.

| Marker | Application | Type | Forward primer | Reverse primer | Amplicon size | Reference |
| --- | --- | --- | --- | --- | --- | --- |
| pfdhfr | ONT | Full length | TATTCCCAAATAGCTAGTTC | AATTTTGTCATCATTTGTTC | 2043 bp | ^6^ |
|  | Sanger | Primary | TTTATGATGGAACAAGTCTGC | AGTATATACATCGCTAACAGA | 646 bp | ^4^ |
|  | Sanger | Nested | ACAAGTCTGCGACGTTTTCGATATTTATG | TAGTATATACATCGCTAACAGAAAT | 638 bp | ^4^ |
| pfdhps | ONT | Full length | AGTATCTATATCTAACTAAAAGAAA | ATTAGAGTACTTGACATATAATGA | 2742 bp | ^6^ |
|  | Sanger | Primary | ATTTTTGTTGAACCTAAACGTGCTGTTCA | CTTGTCTTTCCTCATGTAATTCATCT | 756 bp | ^4^ |
|  | Sanger | Nested | TTGAAATGATAAATGAAGGTGCTAGT | CCAATTGTGTGATTTGTCCAC | 709 bp | ^4^ |
| Pfk13 | ONT | Full length | GAAAATCATAAACAATCAAG | CATTCATTTATTATGTTTTTG | 2464 bp | ^6^ |
|  | Sanger | Primary | CGGAGTGACCAAATCTGGGA | GGGAATCTGGTGGTAACAGC | 2097 bp | ^5^ |
|  | Sanger | Nested | GCCAAGCTGCCATTCATTTG | GCCTTGTTGAAAGAAGCAGA | 849 bp | ^5^ |

Table S2. Primer sequences and amplified fragment lengths.

**Supplementary Figures**


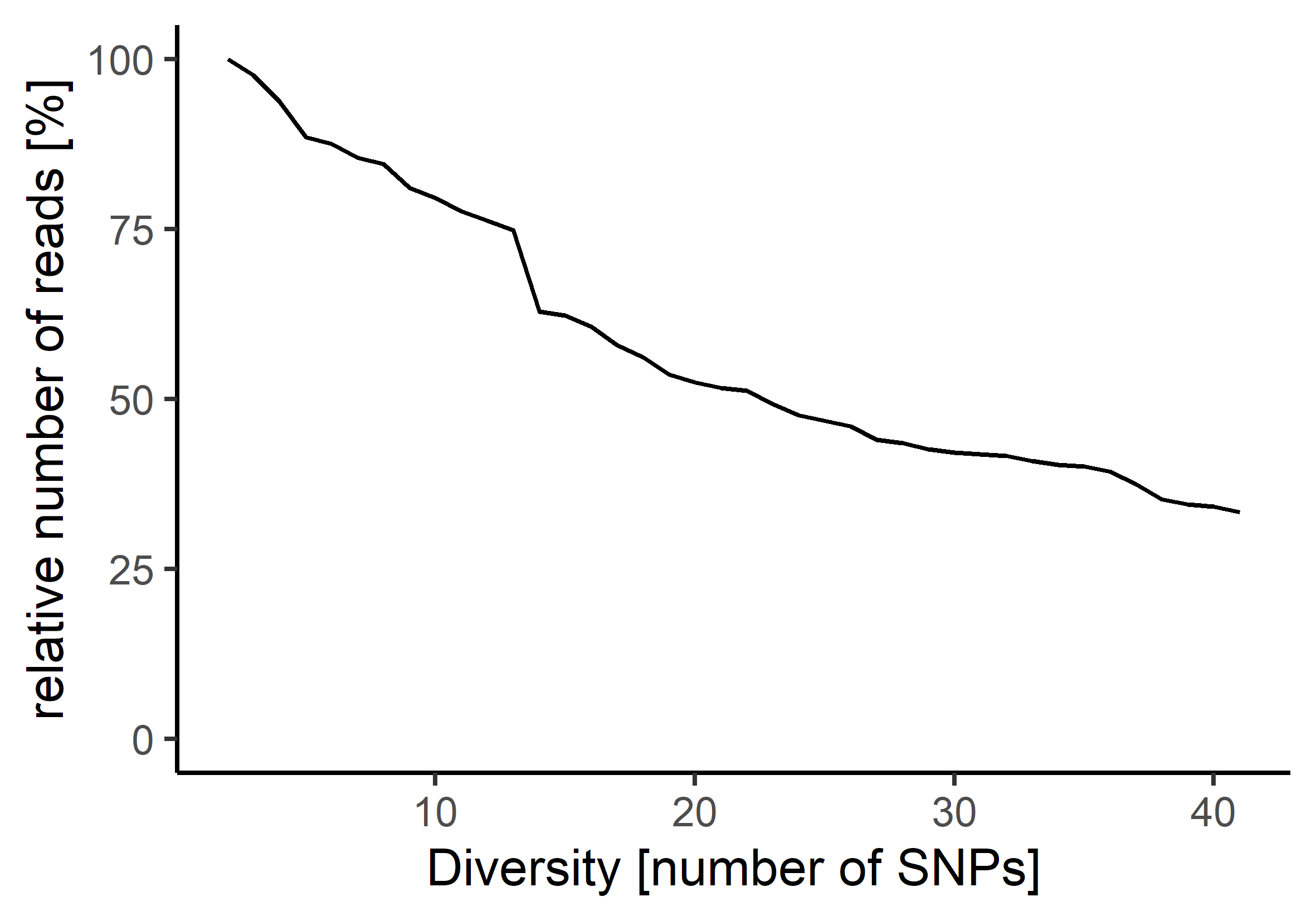


Figure S1. Relative number of reads compared with the number of SNPs used for variant calling. 100% is set at one SNP and corresponds to 5450 reads. To generate this figure, data of the second control experiment was used. The pipeline was run with default parameters 40 times, adding one SNP at a random site with every iteration, storing the mean number of reads over all samples.


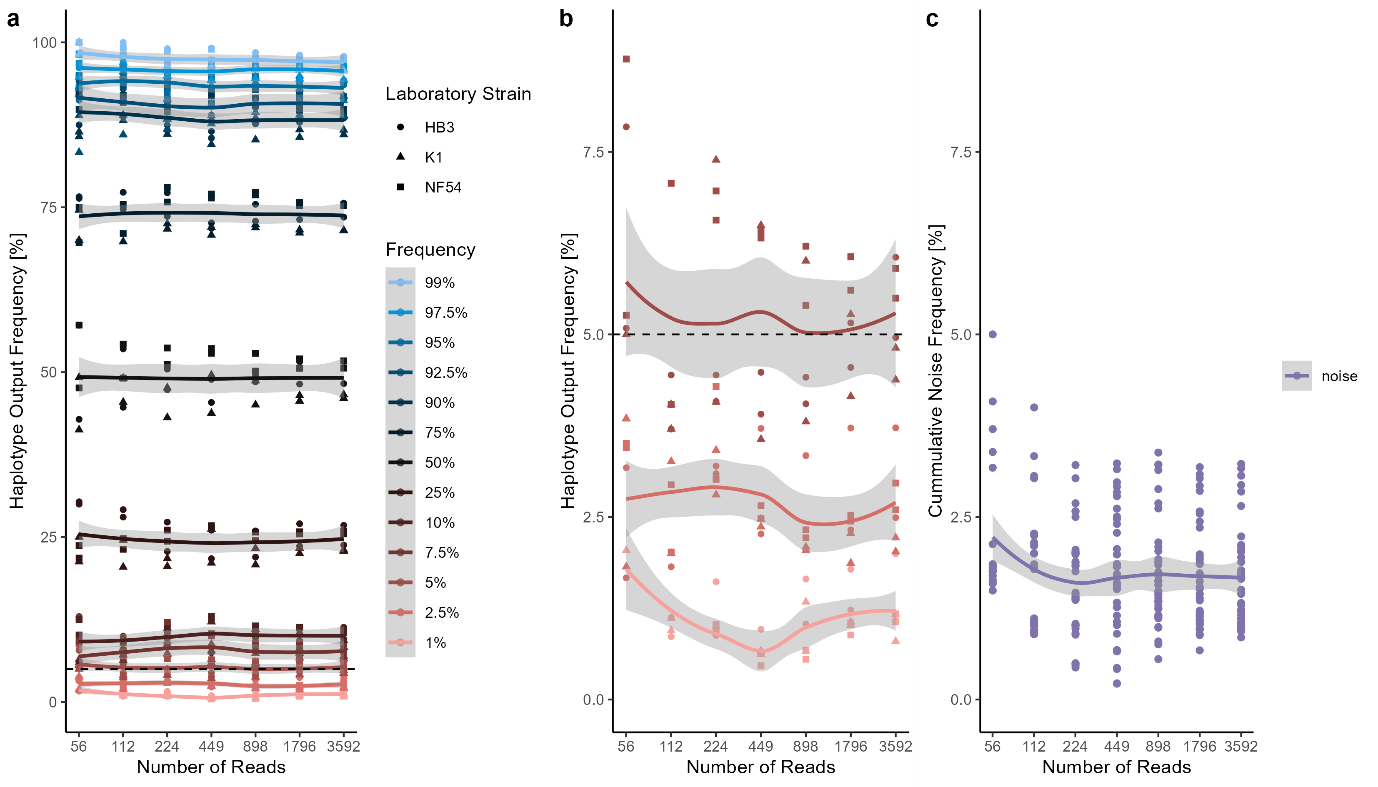


Figure S2: From the first control dataset (in silico mixes of HB3, K1 and NF54 at mixing ratios between 1% and 99%) reads of the *pfdhfr* marker gene were randomly selected for analysis with the PHARE pipeline from 100% of the available reads to 1.56% of available reads. This resulted in a maximum of 3592 reads and a minimum of 56 reads which were used in the final analysis of the pipeline post filtering. In b) a dotted line is drawn at the minimal haplotype frequency of 5%. Haplotypes below would not be considered true haplotypes by the pipeline.


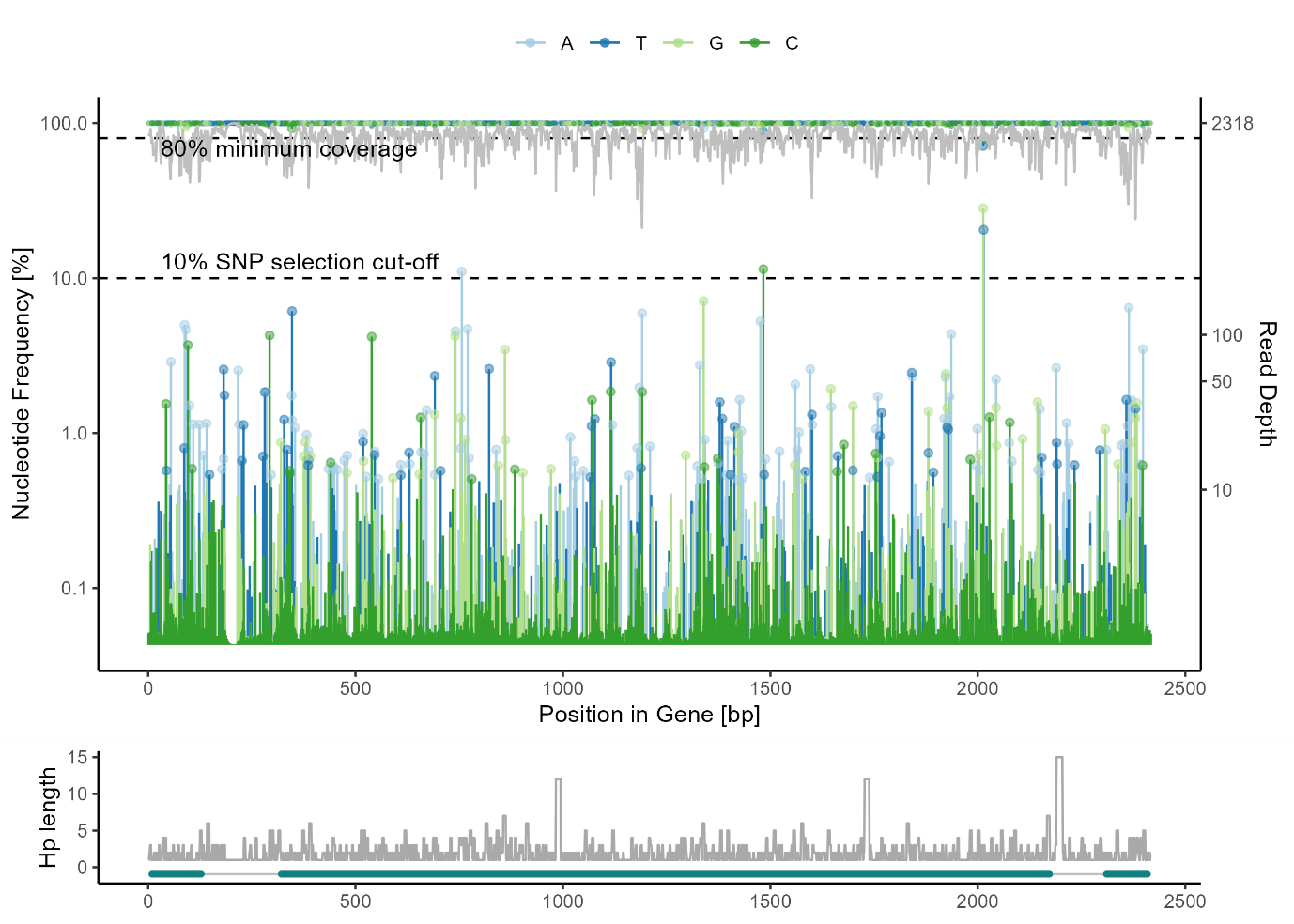


Figure S3. SNP calling using a dataset generated using “fast” basecalling of the 1:1 mix of in vitro culture strains NF54 and Dd2. The nucleotide position is on the x-axis. The read depth is shown in grey and the relative frequency of different bases in the respective colour. The lower dotted line is the SNP selection cut-off, which was set at 10%. The upper dotted line is the coverage minimum which was set at 80%.

Figure S4: Data from *pfdhps* experiment with NF54 and Dd2 in vitro culture strains at different mixing ratios using a minimal quality score of 15 and three different basecalling models of Dorado (v0.4.2). The colours indicate the strain that was inferred based on the haplotypes found by the PHARE pipeline (436f/613s [fs] in Dd2 and S436/A613 [SA] in NF54). Grey dots represent additional haplotypes that are considered sequencing artefacts. A minimum haplotype frequency of 5% (dotted black line) was set to distinguish true haplotypes from noise


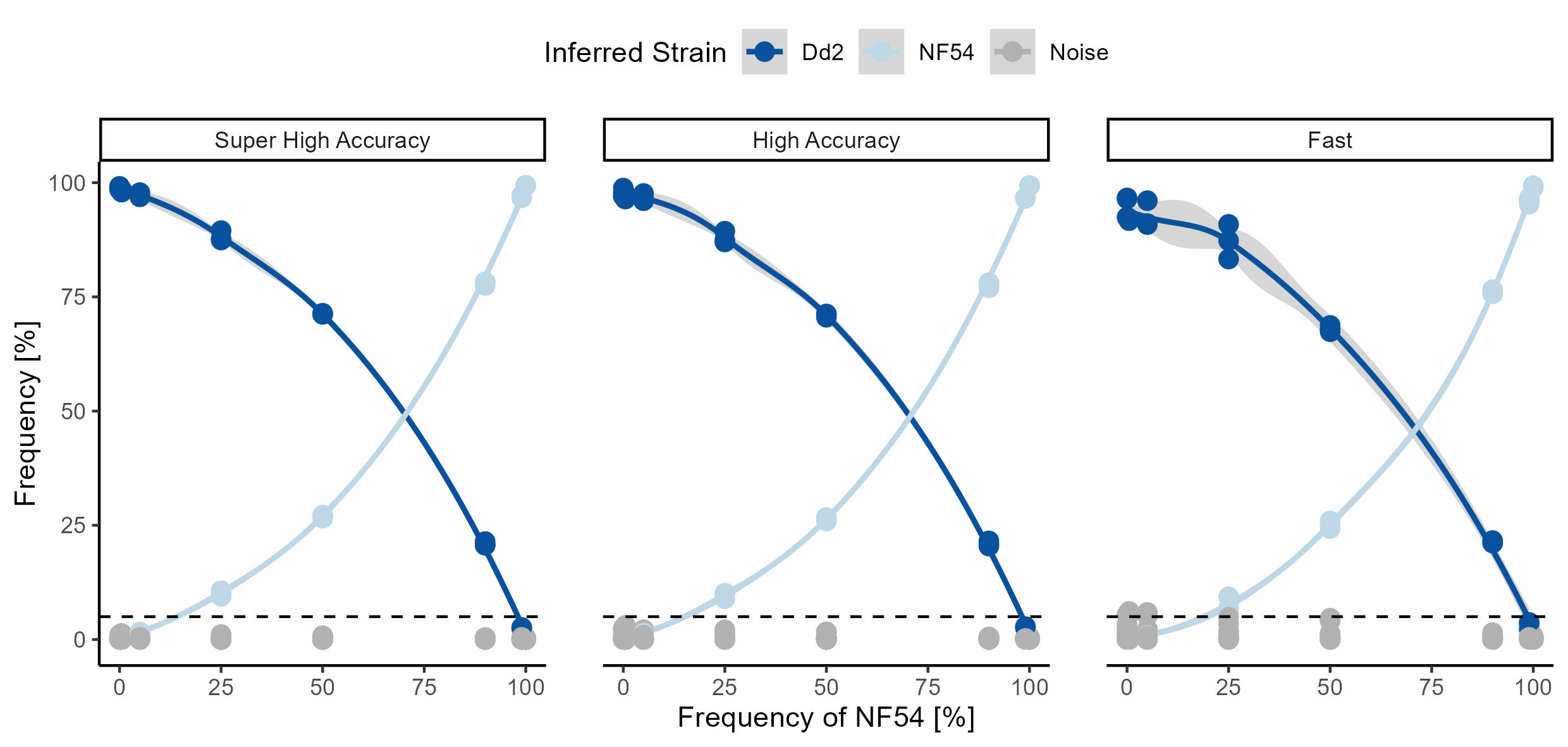


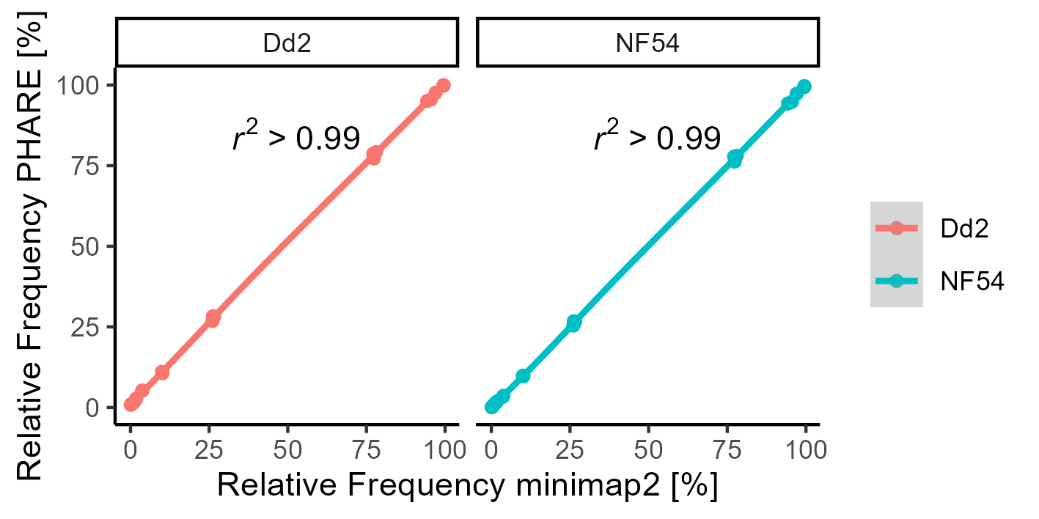


Figure S5. Relative frequencies of the two clones, NF54 and Dd2 when using the PHARE pipeline compared to mapping reads to the corresponding references using minimap2.

Figure S6: Comparison of nano-rave and PHARE for SNP calling with 196 samples collected in Ghana (ENA study accession PRJEB60231) ^7^. Mutant corresponds to >80% mutant and no other haplotype above 5%. Major mutant corresponds to >60% mutant and >5% wild type. Mixed corresponds to both mutant and wild type haplotypes at frequencies of 35% to 60%. Major wild type corresponds to >60% wild type and >5% mutant. Wild type corresponds to >80% wild type and no other haplotype above 5%. Samples with insufficient sequencing coverage are marked unavailable (grey), six samples in *dhps* and 2 samples in *crt*.


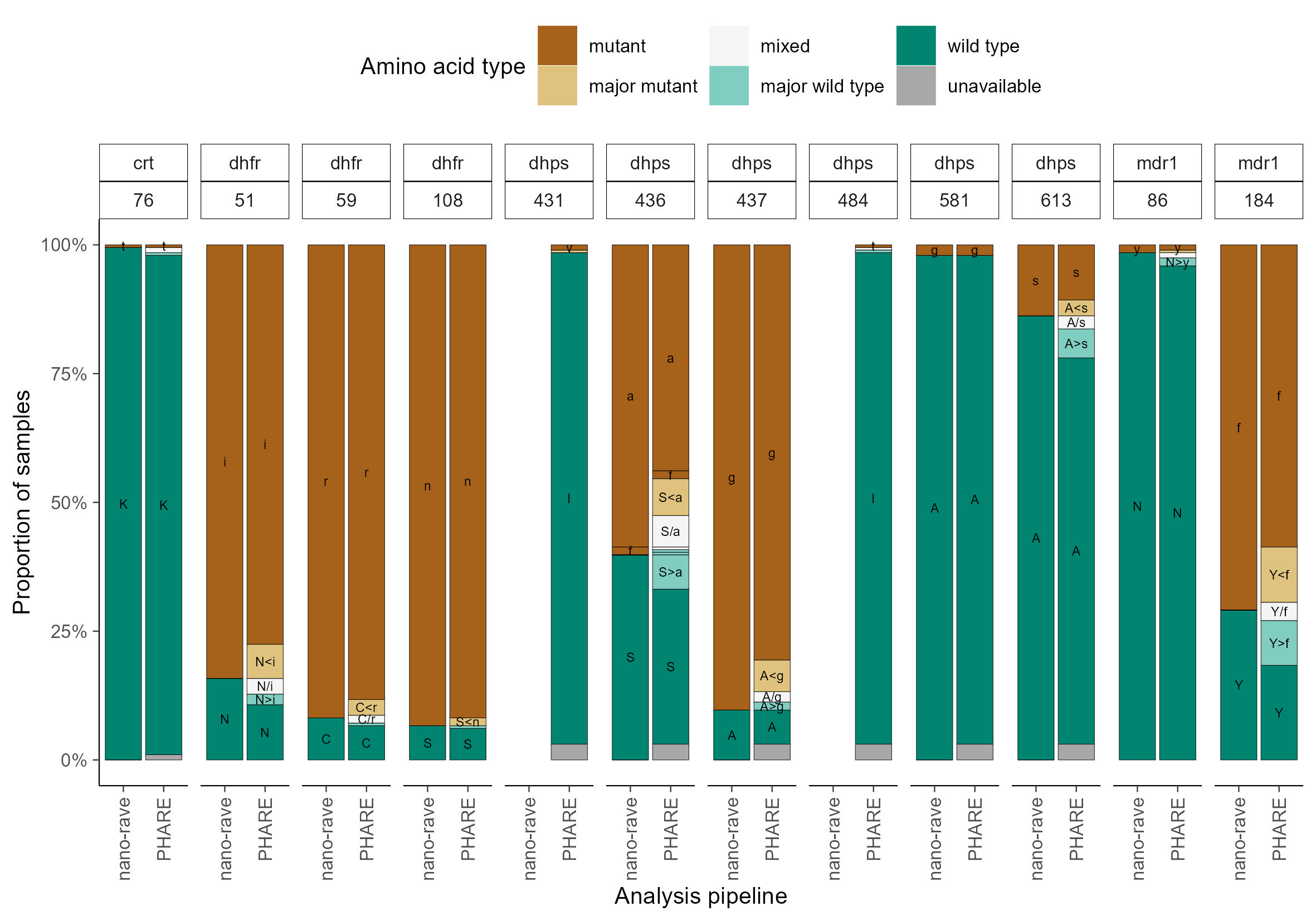


**References**

**1**. Schindler, T., Deal, A. C., Fink, M., *et al.* A multiplex qPCR approach for detection of pfhrp2 and pfhrp3 gene deletions in multiple strain infections of Plasmodium falciparum. *Sci. Rep.* 2019; **9**, 1–10.

**2**. Guirou, E. A., Schindler, T., Hosch, S., *et al.* Molecular malaria surveillance using a novel protocol for extraction and analysis of nucleic acids retained on used rapid diagnostic tests. *Nat. Sci. Reports* 2020; **10**, 1–14.

**3**. Zainabadi, K., Adams, M., Han, Z. Y., *et al.* A novel method for extracting nucleic acids from dried blood spots for ultrasensitive detection of low-density Plasmodium falciparum and Plasmodium vivax infections. *Malar. J.* 2017; **16**, 1–11.

**4**. Chugh, M., Scheurer, C., Sax, S., *et al.* Identification and Deconvolution of Cross-Resistance Signals from Antimalarial Compounds Using Multidrug-Resistant Plasmodium falciparum Strains. *Antimicrob. Agents Chemother.* 2014; **59**, 1110–1118.

**5**. Ariey, F., Witkowski, B., Amaratunga, C., *et al.* A molecular marker of artemisinin-resistant Plasmodium falciparum malaria. *Nat. 2013 5057481* 2013; **505**, 50–55.

**6**. Runtuwene, L. R., Tuda, J. S. B., Mongan, A. E., *et al.* Nanopore sequencing of drug-resistance-associated genes in malaria parasites, Plasmodium falciparum. *Sci. Rep.* 2018; **8**, 1–13.

**7**. Girgis, S. T., Adika, E., Nenyewodey, F. E., *et al.* Drug resistance and vaccine target surveillance of Plasmodium falciparum using nanopore sequencing in Ghana. *Nat. Microbiol.* 2023; **8**, 2365–2390.
